# Psychological and physiological effects of applying self-control to the mobile phone

**DOI:** 10.1371/journal.pone.0224464

**Published:** 2019-11-04

**Authors:** David M. Markowitz, Jeffrey T. Hancock, Jeremy N. Bailenson, Byron Reeves

**Affiliations:** 1 School of Journalism and Communication, University of Oregon, Eugene, Oregon, United States of America; 2 Department of Communication, Stanford University, Stanford, California, United States of America; Radboudumc, NETHERLANDS

## Abstract

This preregistered study examined the psychological and physiological consequences of exercising self-control with the mobile phone. A total of 125 participants were randomly assigned to sit in an unadorned room for six minutes and either (a) use their mobile phone, (b) sit alone with no phone, or (c) sit with their device but resist using it. Consistent with prior work, participants self-reported more concentration difficulty and more mind wandering with no device present compared to using the phone. Resisting the phone led to greater perceived concentration abilities than sitting without the device (not having external stimulation). Failing to replicate prior work, however, participants without external stimulation did not rate the experience as less enjoyable or more boring than having something to do. We also observed that skin conductance data were consistent across conditions for the first three-minutes of the experiment, after which participants who resisted the phone were less aroused than those who were without the phone. We discuss how the findings contribute to our understanding of exercising self-control with mobile media and how psychological consequences, such as increased mind wandering and focusing challenges, relate to periods of idleness or free thinking.

## Introduction

Mobile phones are central to social and professional life in the networked age. For example, meeting a friend at a concert may require purchasing tickets with the phone, a texting conversation or call to arrange the meeting location, and requesting a taxi through a mobile application to arrive at the venue. Despite the relevance and pervasiveness of the mobile phone in everyday settings, researchers often debate the costs and benefits of using the device. Experiments and field studies find that the mobile phone can add social and psychological value to users. For instance, high-risk, low-income youths use cell phones to access emergency help, prevent crime, and mobile devices offer psychological reassurance to bring friends and relatives closer [1]. Mobile phone use can also positively impact civic engagement at scale [2], as phones can be used to organize protests [3] and communicate with distant others that would have been otherwise impossible.

On the other hand, some media theorists [4,5] argue that mobile phone use may be problematic because such devices can negatively affect social and psychological well-being. For example, evidence suggests that high phone dependency individuals have high rates of anxiety, social dysfunction, and insomnia [6]. Interpersonal communication research finds that the mere presence of a phone can negatively influence the quality and closeness of a face-to-face conversation [7]. Finally, frequent mobile phone use can make people vulnerable to negative outcomes, such as interpersonal isolation, because activities on mobile media (e.g., games, social feeds) can make pulling away from screens a challenge [4].

Clearly, there are instances when mobile phone use is beneficial or detrimental to psychological outcomes and social interactions. Little experimental research, however, has considered other common situations with the mobile phone, such as *resisting* the use of the device, that may produce psychological and physiological consequences as well. Exercising self-control with mobile media is common due to social norms (e.g., not using the phone during a meeting), limitations with the technology (e.g., loss of battery or signal), or safety concerns (e.g., not using the phone while driving). In these situations, people must set aside their desire for the technology or communication behavior with technology and use willpower to focus psychological resources elsewhere [8].

What are the psychological and physiological costs of exercising self-control with the mobile phone? This question is central to our study, as we examine how people feel psychologically and how their body responds to resisting the mobile phone relative to being without the device or using it.

Our experiment has two primary objectives. First, we test if exercising self-control by resisting the mobile phone leads to changes in how people perceive their psychological experience and how their body responds, physiologically. This approach is timely because prior work has largely examined how time spent using media (e.g., web-surfing, playing video games) is often negatively correlated with self-control and therefore considers media use a consequence of reduced willpower [9]. We examine another possibility where exercising self-control with the mobile phone *causes* psychological and physiological consequences relative to mobile phone use or absence.

Embedded in this first objective is another goal of replicating and extending related work. A host of research suggests that several psychological processes (e.g., enjoyment, concentration abilities) are negatively impacted by a lack of external stimulation and people report that having time to “just think” is less positive than having something to do [10,11]. We explore if resisting the mobile phone amplifies the consequences of this well-documented challenge, and how being without the mobile phone changes an individual’s perceptions and physiological arousal relative to using the device. Drawing on literature from psychology and communication research, we dissect each of these experiences with mobile media (e.g., self-control, aloneness, and use) to understand how they might affect the way people think and feel.

### Delaying gratification and self-control

The idea that resisting the mobile phone might lead to important psychological and physiological consequences is rooted in a tradition of research on delaying gratification [12]. In a classic experiment, Mischel and Ebbesen demonstrated how there are downstream benefits for children who postpone a temptation better than others [13]. The authors randomly assigned children to receive an immediate, less preferred reward such as pretzels, or a delayed, more preferred reward such as cookies. In one condition, children were left in a room without any reward. In a second condition, children had both rewards available to them. In the final two conditions, either the less preferred or more preferred reward was present, only. Mischel and Ebbesen [13] observed that when children had any reward present, they waited for less time and had more difficulty delaying gratification than if no reward was present. The authors argued that the saliency (e.g., the treats were directly in front of the children), draw, and anticipation of the reward were cognitively consuming for the children and applying self-control to temptations, in general, is difficult.

Mischel’s overarching paradigm, the Marshmallow Test, found that children have short-term difficulties delaying gratification and the ability to delay is highly correlated with long-term measures such as intelligence (e.g., better delayers have higher standardized test scores than worse delayers) and health (e.g., better delayers have lower body mass indices than worse delayers; [14]). These findings, and over forty years of research on delaying gratification and self-control, suggest that there are benefits to successfully resisting temptation [8,14,15]. It is therefore important to understand the self-control demands with desirable stimuli that include objects in today’s media environment, such as the mobile phone.

Self-control is difficult because individuals must remain vigilant in overriding their natural behaviors (e.g., using their phone in a period of free time) and then acting in a manner that is different from their ordinary behavior (e.g., resisting their phone). Prior work suggests that self-control failures occur because people focus less on self-control and more on gratifying desires when a temptation is present. Exercising self-control stifles the “monitoring system for getting self-control started” and individuals are less likely to recognize cues suggesting that willpower is needed [15] (p. 456). People should therefore report psychological challenges and costs (e.g., increased focusing challenges) when the mobile phone is in front of them—but they must resist it—since this act conflicts with social and psychological goals.

### Aloneness and just thinking

While decades of self-control research suggests that resisting temptation has downstream social and psychological consequences [8,16], evidence also suggests that people who sit idly and entertain themselves with their thoughts report less positive psychological outcomes (e.g., less enjoyment, more concentration difficulty, and more mind wandering) compared to people who have external stimulation. For example, 11 studies by Wilson and colleagues [11] found that people would rather have something to do instead of thinking freely (see also [17]), even if the self-inflicted stimulus is negative such as electric shocks.

More recent research suggests that free thinking—defined here as a period of being alone with one’s thoughts, without external stimulation, and without a cognitive thinking aid—is a challenge for people unless they have a specific goal or topic to think about. For example, Alahmadi and colleagues [10] observed the impact of motivation on periods of aloneness. Across four studies, the authors gave participants instructions to “think about whatever you want” or they wrote down enjoyable items to think about (e.g., a family event) and then engaged in a similar thinking task ([10], p. 547). Without explicit topic-related instructions, participants rated the experience as less enjoyable and their mind wandered more than those who were told to think about a positive event. This evidence, and data from similar studies, have been supported at scale [18–21], reinforcing the general theory that people who have something to think about often report feeling psychologically better than people who are free-thinking, idle, or alone without external stimulation.

Why is being alone with one’s thoughts psychologically less positive than having something to do? There are several possible reasons outlined by Westgate and colleagues [18]. Chief among them is the idea that giving people a topic to think about (a “thinking aid”) may reduce cognitive demands on the participant relative to not giving topic or thought instructions, therefore making the experience more enjoyable. Free thinking, in general, is not universally challenging, but unfocused and non-directed thinking (or “just thinking”) is less positive than “doing,” or performing “everyday solitary activities” [19] (p. 2). Periods of aloneness should therefore elicit less positive responses in people relative to having a form of external stimulation. Typical outcomes of aloneness and free thinking include less reported enjoyment, more concentration difficulty, and more mind wandering [10,11,18]. While enjoyment offers a relatively straightforward prediction (e.g., people typically have a less positive response to being alone with their thoughts), concentration difficulty and mind wandering are less clear.

Consistent with prior work [10,11,18,19], concentration difficulty and mind wandering are considered negative traits in this study because they suggest a lack of attention and focus [22]. Early but still relevant daydreaming research by Singer [23] suggests that there are three types of mind wandering [24]. Mind wandering can be considered a period of creative and playful thought (“positive constructive daydreaming”), a period of aggression and obsessive hostility that focuses on others (“guilty-dysphoric daydreaming”), or a time characterized by increased concentration difficulty on a thought or task (“poor attentional control”). To remain consistent with prior research evaluating the psychological effects of free thinking versus periods of external stimulation [10,11,18], the present study conceptualizes mind wandering as a lack of attentional focus on a thought or task. This assumes that non-directed thinking is generally associated with fewer positive outcomes than directed thinking or periods with external stimulation.

We use concentration difficulty and mind wandering as complementary measures in our study to evaluate how periods of aloneness impact psychological outcomes. This conceptualization is also in line with other research [10], which suggests that free thinking and enjoying one’s thoughts have competing psychological effects. An experience that induces high rates of concentration difficulty and mind wandering is therefore less enjoyable, and “to the extent that people succeeded in concentrating, they experienced less mind wandering and greater enjoyment” [10] (p. 556). We therefore follow and build on existing literature by comparing aloneness (a period of free thinking) to a period of self-control and mobile phone use.

### Periods with external stimulation

Experiments by Wilson and colleagues generally suggest that idleness, aloneness with one’s thoughts, and periods of free thinking are psychologically less positive than periods of directed thinking or external stimulation [10,11,18,19]. Recall, people report more favorable psychological outcomes (e.g., more enjoyment, better focus and concentration) when they have something to do (e.g., read a book, listen to music, go online) rather than just thinking. By contrast, then, periods with external stimulation such as using the mobile phone should be psychologically beneficial and cause similar psychological changes compared to aloneness.

People often enjoy using mobile media because the phone provides an opportunity to access social outlets when information-seeking and entertainment are particularly salient desires [9], it allows people to form routines with media [25], and the phone makes people and information always available [26]. External stimulation, in general, is psychologically beneficial because it focuses cognitive resources [10,18]. We expect positive outcomes (e.g., more enjoyment, better concentration abilities, less mind wandering) for people with any form of external activity (e.g., “doing”) compared to “just thinking” and exercising self-control.

### Predictions

The prior rationales motivate a three-condition test of how mobile phone experiences, such as having to resist, be without, or use the device, affect psychological and physiological outcomes. Resisting the mobile phone should lead to psychological and physiological consequences because the device is a hub for access to social and professional connections, activities, and entertainment [27]. The desire to use media and act on what they afford are pervasive. A week-long study pinged participants at seven instances per day, asking them to record their current desire (or one that they just experienced) and if they attempted to resist the reported desire [28]. The desire for media use (e.g., social networking sites, attending to email, using the Internet) resulted in one of the highest self-control failures (e.g., 42% of media desires were enacted despite resisting, more than sex and drugs). Other work [29] surveyed college students’ media use and found a negative relationship between self-reported self-control (e.g., rating agreement with statements such as “I am good at resisting temptation”) and time spent using leisure media. Participants also reported several motivations for Internet use, and only self-control inversely predicted the amount of time that students would use social networking sites. Therefore, self-control with media may have nontrivial moderating effects on psychological dynamics.

On the other hand, experiments following the Wilson and colleagues tradition [10,11,18,19] provide a valuable foundation to predict how people without their device and who are provided with “just thinking” instructions will report psychological reactions to aloneness. Most of the evidence following Wilson and colleagues’ procedures suggests that people face enjoyment and concentration challenges when they “just think” versus if they have something to do. While the severity of this psychological challenge has been debated [30], overwhelming evidence supports the general theory that free thinking and being without a cognitive aid is perceived as psychologically less positive or even neutral relative to a period with external stimulation [10,11,17–19,31].

We therefore presuppose that when the mobile phone is placed in front of the participant but he or she is resisting the device, this task replicates the treatment condition of the Marshmallow Test experiments. Children had difficulty resisting the marshmallow because the temptation was directly in front of them. People have a natural gravitation towards satisfying desires (e.g., using the phone) rather than resisting desires [8], suggesting that self-control with media will be difficult. Therefore, an experience without the phone should be challenging but less distressing than an experience with the phone but self-control is exercised. This “out of sight, out of mind” experience was observed in the Marshmallow Test experiments, as children who closed their eyes or looked away from the marshmallow could exercise self-control better than children who stared at the marshmallow [16] (p. 31).

Participants who resist their phone should experience lower levels of enjoyment, more concentration difficulty, and more mind wandering than participants who use the phone or sit alone without the device. When people are using the phone, their desires are satisfied and their self-regulation goals are not in conflict.

H_1_: Participants who resist their phone will report less enjoyment, more concentration difficulty, and more mind wandering relative to participants who sit without their phone or who use their phone.

To evaluate if exercising self-control affects automatic responses to temptation, electrodermal activity (EDA), also known as skin conductance, captured levels of physiological arousal. EDA is measured by passing a current through two electrodes that touch the skin [32]. Skin conductance corresponds to changes in sweat levels and suggests the activation of fight-or-flight responses [33]. This “secretory theory” argues that sweat glands are activated under periods of high arousal and less activated under periods of low arousal [32,34]. Crucially, EDA varies with the intensity of the activation to physiological systems. For example, highly emotional and arousing images produce greater skin conductance levels relative to neutral images [35] and people tend to experience the greatest electrodermal responses to highly uncomfortable experiences.

Communication frameworks, such as the Limited Capacity Model of Motivated Mediated Message Processing (LC4MP [36,37]), provide an important theoretical foundation to investigate the relationship between EDA, the mobile phone, and periods of aloneness, self-control, and use. Though the LC4MP typically conceptualizes how human motivational systems respond to mediated messages, the model is still relevant to understand how psychophysiological systems relate to media experiences, in general [38]. Lang [36,37] proposes four main arguments in the LC4MP: (1) our capacity to encode, store, and retrieve information in a mediated world is limited, (2) humans have a motivational system that facilitates appetitive (approachable) or aversive (avoidance) reactions to media, (3) media experiences are multidimensional, involving a host of sensory inputs (e.g., sight, sound) and modes (e.g., text, images, devices), and (4) human behavior is dynamic and changes over time.

Two LC4MP propositions are most relevant to the current investigation. The idea that people may have appetitive or aversive responses to media depending on the experience, plus the dynamic nature of psychophysiological systems over time, motivate an investigation into how people process self-control with the mobile phone, aloneness, and mobile phone use. In this way, physiological arousal can be used as an indicator of “embodied mental processes” [39]. Since prior work has applied tenets of the LC4MP to a host of media experiences beyond messaging (e.g., multitasking [38,40,41]), we use this theory to motivate our test of how people will respond psychologically and physiologically to three common experiences with mobile media.

Evidence diagnosing the relationship between EDA and media experiences is complex with mixed results from fields of communication and psychology. For example, some people display greater physiological arousal when absent from their phone compared to having their device. In a within-subjects design, Clayton and colleagues [42] had participants complete word puzzles with or without their cell phone present. When a participant’s phone was knowingly placed in a different room and they heard it ring, increased heart rate, blood pressure, and anxiety levels were detected relative to when the same person had their phone in the environment. On the other hand, suppressing impulses (e.g., engaging in self-regulation) can be highly arousing as well. Gross [43] randomly assigned participants to one of three conditions (e.g., cognitive reappraisal, thought suppression, no instructions) and had them watch a highly negative and arousing film of an amputation. Participants who suppressed their emotions had greater arousal than those who watched the movie or reappraised its content.

Thought suppression and self-regulation, in general, have negative consequences that are well-documented in social psychology. In a classic paper by Wegner and colleagues, participants who resisted the thought of a white bear experienced a rebound effect [44], where people who suppressed their thoughts then reflected on the white bear more than those who were not given thought regulation instructions. Together, based on extant evidence, there are two possible physiological outcomes for the relationship between self-control and the mobile phone: (1) being without the phone is more arousing than using it [42], and (2) resisting temptation and exercising self-control with the phone is more arousing than using it [43,44]. Consistent with these plausible outcomes, we predict contrasting hypotheses.

H_2a_: Physiological arousal will be greatest for participants who are without their phone, relative to those who use or resist their phone.
H_2b_: Physiological arousal will be greatest for participants who resist their phone, relative to those who use or are without their phone.

## Method

All preregistered hypotheses and data for this study are stored on the Open Science Framework (OSF: https://osf.io/64tdm/). Preregistration occurred in early January 2017 and data collection started two weeks after the preregistration.

### Power

We collected data in waves to ensure a fair chance of detecting an effect based on power recommendations from prior research. The experimental paradigm from Wilson and colleagues [11] (Study 8) required 30 participants across two conditions to observe a large effect (*r* = .67). Prior replication attempts required 18 participants per condition to detect 75% of the original effect, powered at 90% (α = .05, two-tailed) [45]. Our experiment follows this power recommendation (54 participants total; 18 per condition), defined as Wave 1 of data collection.

Consistent with Holzmeister and colleagues [45], a second wave of data collection to detect 50% of the original effect size (powered at 90%) commenced after several Wave 1 effects were not statistically significant (see Wave 1 Results on the OSF). We used the second wave to test whether the null effects were indeed genuine or perhaps an artifact of an underpowered research design.

The power recommendation based on the two-condition Wilson and colleagues [11] (Study 8) design required 42 participants per cell in Wave 2. Our three-condition design required a pooled sample size of 126 participants. Results for the pooled data are provided below. Our preregistration plan was carried out for the entire pooled dataset of participants.

### Participants and exclusion criteria

Participants were students at Stanford University and compensated with course credit for their time in the experiment. The study was approved by Stanford University’s Institutional Review Board (protocol ID# 35431), with written consent obtained, and advertised as a “Thinking and Concentration” study to not raise suspicion or focus attention on mobile phones.

Several participant exclusion criteria were also predetermined. Data were excluded if: (a) participants terminated the experiment by exiting the room, (b) participants failed to answer all questionnaire items, (c) parts of the study’s infrastructure faced technical issues, or (d) a participant held strong views toward media during or after the experiment. Only one participant was excluded, who fit the fourth criterion.

The final pooled sample had 125 participants (74 females). Participants were mainly Asian/Pacific Islander (52/125; 41.6%) and white (39/125; 31.2%), with mixed race (22/125; 17.6%), Hispanic/Latino (7/125; 5.6%), and African American (5/125; 4.0%) orientations being less represented in the sample. Race, [(χ^2^(8) = 10.40, *p* = .24], and age were not significantly different across the three conditions [(*M* = 20.85 years, *SD* = 1.93 years); *F*(2, 122) = 1.34, *p* = .27]. Gender was also evenly distributed across conditions, [(χ^2^(2) = 0.80, *p* = .67]. Therefore, this evidence suggests that random assignment was indeed successful.

### Procedure

Participants were randomly assigned to one of three conditions: (1) *phone resist*, treatment condition, (2) *no phone*, control condition, or (3) *phone use*, control condition. Before the experimenter described the study’s procedure, participants left all belongings and accessories (except for the mobile phone in relevant conditions) in an adjacent control room. First, the experimenter explained how physiological data would be measured with a tracker. A pretest revealed that having the tracker placed on the participant’s wrist produced low and unreliable electrodermal readings. Therefore, participants were instructed to place their index and middle finger of their non-dominant hand on the tracker and keep them in place for the entire experiment [46]. This method was successful at achieving reliable readings. The experimenter then had the participant sign a consent form and provide demographic data (see Supplementary Materials on the OSF). The experimenter was consistent across all participants.

In the *phone resist* condition, participants sat in an unadorned room for 6 minutes, a time called the “thinking period.” The experiment length was not revealed to any participant across conditions. Participants in the phone resist condition placed their cell phone face down on the desk, but on silent mode with the power on. They were told to entertain themselves with their thoughts, not fall asleep, remain seated, and not use their phone [11]. Exact wording for the instructions to participants in this condition are on the OSF.

In the *no phone* condition, the participant’s phone (and watch, where applicable) was placed in a separate room. Participants were told to remain seated, entertain themselves with their thoughts, and to not fall asleep.

In the *phone use* condition, participants were instructed to entertain themselves with one or more activities on their phone (e.g., watching video clips, reading, or surfing the web) during “the experimental period.” Participants were told that they could switch from one mobile phone activity to another, with the goal of “finding something enjoyable to do.” They were further instructed not to call or text during the experimental period, “because the goal of the experiment is to find something entertaining that you like to do alone” [11]. This qualification was included to directly match the task instructions from prior work.

For all conditions, after six minutes elapsed, the experimenter administered the dependent measures and questionnaires. After the participant submitted responses to the questionnaires, each participant was debriefed by the principal investigator. Participants were videotaped to surveil room activity and ensure compliance with the procedures. As required by our ethics board approval, participants were aware that they were being videotaped. Only one participant in the *phone resist* condition touched but did not use the device. Still photos of participants are located in a supplementary figure on the OSF (https://osf.io/64tdm/).

### Measures

#### Enjoyment

Participants first answered three questions about how much they enjoyed the thinking or experimental period. Text in brackets are wording changes for participants in the phone use condition, because their instructions did not mention a “thinking period.”

The first question asked, “How enjoyable was [the thinking period/the experience] you just had?” from (1) Not enjoyable at all, to (9) Extremely enjoyable. The second question asked, “How entertaining was [the thinking period/the experience] you just had?” from (1) Not entertaining at all, to (9) Extremely entertaining. The third question asked, “How boring was [the thinking period/the experience] you just had?” from (1) Not boring at all, to (9) Extremely boring. Consistent with prior work [10,11], these three questions were combined into a composite measure of enjoyment [(*M* = 5.52, *SD* = 1.77), Q1 = 4.33, *Mdn* = 5.67, Q3 = 6.67; Cronbach’s α = 0.87] by averaging the three measures (boredom was reverse scored for the creation of the index, only).

#### Concentration difficulty

Participants were asked, “How difficult was it for you to concentrate [on the thing that you were thinking about/during this experience]?” from (1) Not at all, to (9) Very much [11]. The average level of concentration difficulty was below the midpoint [(*M* = 3.55, *SD* = 1.84), Q1 = 2.00, *Mdn* = 3.0, Q3 = 5.0].

#### Mind wandering

Participants were asked, “To what extent did you find that your mind wandered during [the thinking period/this experience]?” from (1) Not at all, to (9) Very much [11]. The average level of mind wandering was close to the midpoint [(*M* = 5.35, *SD* = 2.08), Q1 = 4.0, *Mdn* = 5.0, Q3 = 7.0].

#### Positive and negative affect

Participants completed the Positive and Negative Affect Schedule (PANAS) as a measure of general mood [47]. Participants rated how well positive, [(*M* = 22.54, *SD* = 7.71), Q1 = 17.0, *Mdn* = 21.0, Q3 = 28.0], and negative adjectives, [(*M* = 14.57, *SD* = 5.38), Q1 = 11.0, *Mdn* = 13.0, Q3 = 16.0], described their current mood from (1) Very slightly or not at all, to (5) extremely. The positive (Cronbach’s α = 0.90) and negative dimensions (Cronbach’s α = 0.88) were highly reliable.

#### Mobile phone perceptions, use, and intensity

To understand if perceptions of the mobile phone and its importance in everyday life were different across conditions, a composite scale of five mobile phone use intensity questions was adapted from prior work [48] (Cronbach’s α = 0.71). Participants rated the following questions on a five-point scale from (1) strongly disagree to (5) strongly agree: (a) The mobile phone is part of my everyday activity, (b) I am proud to tell people that I use my phone daily, (c) Using my phone has become a part of my daily routine, (d) I feel out of touch when I haven’t used my phone for a while, (e) I would be sorry if my phone was unavailable to me for some time. This variable is henceforth referred to as the *mobile phone perceptions* scale.

Participants also reported how many minutes per day they use specific apps or services on their phone (e.g., Facebook, Twitter, email, texting, browsing the Internet, online dating/mobile dating apps, phone calls, YouTube, music apps; Cronbach’s α = 0.76). We refer to this measure as the *mobile phone use* scale.

Though we analyzed these measures separately, they were also correlated, (*r* = .20, *p* = .029). We therefore formed a composite dimension called *mobile phone intensity* by adding the two standardized scales. These measures also loaded onto a single factor (λ = 1.20), accounting for 59.78% of the variance.

#### Physiological arousal

Skin conductance was measured with the Empatica E4 tracker [(*M* = 2.44 raw μS, *SD* = 1.82 raw μS), Q1 = 1.11 raw μS, *Mdn* = 1.98 raw μS, Q3 = 3.26 raw μS], collected at a sampling rate of 4 hertz per second, and measured in microSiemens (μS). Data from 15 participants were excluded from the EDA analyses because of faulty skin conductance recordings (e.g., the tracker failed to record data; *n* = 5) and extreme statistical outliers (e.g., participants whose average EDA readings were more than 3 times above or below the height of the boxes in a Box Plot analysis; *n* = 5, including the one participant who was removed because of the exclusion criteria mentioned above). Based on emerging standards for measuring skin conductance [32,46,49], five participants were also excluded because their (raw) average EDA rates registered less than 0.5 μS (final *N* = 111 for EDA analyses).

#### Preprocessing

Skin conductance data for each participant were divided into 15-second increments and averaged within each epoch. Consistent with prior work, we then transformed the data by subtracting the tonic (baseline) EDA, defined as the average skin conductance one minute before the start of the experimental period (e.g., when the participant filled out the demographic survey), from the raw experimental period EDA [32,50–52]. For example, if the average experimental period EDA within the first 15-second epoch registered 1.41μS and the average tonic EDA was 1.50 μS, the -0.09 μS difference reflects the manipulation’s effect on arousal level. This participant would be less aroused during the experimental period relative to baseline because the sign is negative.

The demographic questionnaire was administered with pencil and paper because participants kept their fingers on the Empatica electrodes to gather baseline measurements of arousal. This non-invasive data collection method was consistent with best practices outlined by Boucsein [53]. Collecting this baseline data and having participants type with one hand was unwieldy and could possibly increase arousal if the task became difficult. All other questionnaires were administered online through a Qualtrics survey interface.

## Results

The data were analyzed using one-way Analysis of Variance models (see Table 1). For the self-report psychological variables, we planned three statistical tests (i.e., the overall enjoyment index, concentration difficulty, and mind wandering) to measure mean differences across conditions. Three exploratory tests were also performed for the enjoyment index items to evaluate if univariate patterns (enjoyable, entertaining, boring) were consistent with the overall effect. We then planned five statistical tests to measure mobile phone perceptions data (i.e., mobile phone perceptions, use, and intensity) and emotional responses (positive and negative PANAS measures) across conditions. Multiple comparison tests are *Bonferroni*-corrected in the main text to reduce the likelihood of Type I errors. S1 Table contains multiple comparisons effects across all measures.

**Table 1:** Means and standard errors for self-report and arousal variables by condition.

	Phone resist		No phone		Phone use				
	(*n* = 42)		(*n* = 42)		(*n* = 41)				
	*M*	*SE*	*M*	*SE*	*M*	*SE*	*F*	*p*	η^2^_p_
Enjoyment	5.30	0.27	5.52	0.27	5.74	0.28	0.63	.53	.01
Enjoyable	5.48	0.29	5.76	0.29	5.66	0.29	0.25	.78	.004
Entertaining	4.33	0.30	4.79	0.30	5.51	0.31	3.80	.025	.059
Boring	3.91	0.33	3.98	0.33	3.95	0.33	0.01	.99	< .001
Concentration difficulty	3.33	0.27	4.31	0.27	3.00	0.28	6.16	.003	.092
Mind wandering	5.76	0.30	6.07	0.30	4.20	0.30	11.22	< .001	.155
EDA	-0.29	0.10	-0.18	0.09	-0.19	0.10	0.44	.645	.008
PANAS Positive	22.48	1.20	22.67	1.20	22.49	1.21	.008	.99	< .001
PANAS Negative	14.24	0.83	15.36	0.83	14.10	0.84	0.69	.51	.011

Enjoyment is a composite variable consisting of the enjoyable, entertaining, and boring measures. The boring scale was reverse scored when computing the enjoyment index. EDA = Electrodermal Activity. EDA data were calculated using a baseline subtraction method, with higher scores representing greater arousal relative to baseline. All multiple comparison tests are provided in-text.

### Pooled Wave 1 and Wave 2 Data: Self-report psychological variables

#### Enjoyment

The effect of condition on the enjoyment index failed to reach significance, [*F*(2, 122) = 0.63, *p* = .53, η^2^_p_ = .01]. We also examined the enjoyable, entertainment, and boring variables separately in an exploratory analysis. Only the entertainment item revealed a significant effect, [*F*(2, 122) = 3.80, *p* = .025, η^2^_p_ = .059]. Participants who resisted their phone viewed the experiment as less entertaining (*M* = 4.33, *SE* = 0.30) than participants who used their phone [(*M* = 5.51, *SE* = 0.31); *p* = .022].

#### Concentration difficulty

The effect of condition on reported concentration difficulty during the thinking or experimental period was significant, [*F*(2, 122) = 6.16, *p* = .003, η^2^_p_ = .092]. Participants who resisted their phone had less difficulty concentrating (*M* = 3.33, *SE* = 0.27) than participants with no phone [(*M* = 4.31, *SE* = 0.27); *p* = .038]. Participants who used their phone also reported less concentration difficulty (*M* = 3.00, *SE* = 0.28) than participants without their phone as well [*p* = .003]. There was no difference in reported concentration difficulty between participants in the phone resist and phone use conditions, [*p* > .80].

#### Mind wandering

The effect of condition on reported mind wandering during the thinking or experimental period was significant, [*F*(2, 122) = 11.22, *p* < .001, η^2^_p_ = .155]. Participants who used their phone (*M* = 4.20, *SE* = 0.30) reported less mind wandering than participants who resisted their phone [(*M* = 5.76, *SE* = 0.30); *p* = .001] and did not have their phone [(*M* = 6.07, *SE* = 0.30); *p* < .001]. There was no difference in mind wandering between participants who resisted and who did not have their phone, [*p* > .80].

Together, H_1_ was partially supported.

#### Mobile phone perceptions, use, and intensity

Participants across conditions did not differ on the *mobile phone perceptions*, *mobile phone use*, or *mobile phone intensity* measures [*F*’s < 2.02, *p*s > .14].

#### PANAS

There were no reported differences in positive affect [*F*(2, 122) = 0.01, *p* = .99, η^2^_p_ < .001] or negative affect [*F*(2, 122) = 0.69, *p* = .51, η^2^_p_ = .011]. This pattern is consistent with prior work that suggests resisting temptation is typically unassociated with changes in positive or negative mood [54].

### Pooled Wave 1 and Wave 2 Data: Physiological arousal

**Fig 1:**
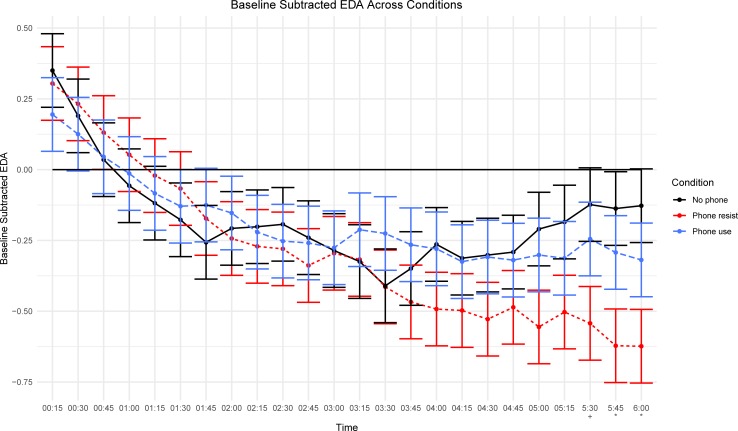
Baseline subtracted EDA across conditions. *N* = 111 participants. Average EDA is calculated between two time points, represented on the X-axis. * *p* < .05. + *p* < .07. Significant differences at each time marker are *Bonferroni-*corrected and represent differences for the no phone and phone resist conditions. Error bars represent 1 Standard Error above and below each sample mean. Values above and below zero represent increased and decreased arousal relative to baseline, respectively.

We used a linear mixed model to compute a Condition (phone resist, no phone, phone use) X Time (24 x 15-second windows) interaction, with a random effect for participant to account for repeated, non-independent observations by the same individual. The Condition X Time interaction was significant, [*F*(46, 2484) = 1.41, *p* = .038], and revealed a decline in EDA for all participants over time (Fig 1). This decline remained robust for those who resisted their phone and EDA eventually flattens for those in the phone use condition.

At approximately the three-minute mark, however, EDA for those in the phone resist and no phone conditions started to significantly diverge. As expected, the time main effect was statistically significant, [*F*(23, 2484) = 13.70, *p* < .001]. The EDA main effect across phone resist (*M* = -0.29 μS, *SE* = 0.10 μS), no phone (*M* = -0.18 μS, *SE* = 0.09 μS), and phone use (*M* = -0.19 μS, *SE* = 0.10 μS) conditions was not significant, [*F*(2, 108) = 0.44, *p* = .645]. The data support H_2a_ over H_2b_ after considering the effects over time.

## Discussion

The majority of academic research evaluating the relationship between media and self-control has considered media use as a *consequence* of reduced or exhausted willpower [9]. This study tested a different possibility, where applying self-control to the mobile phone changes psychological and physiological processes. The data reveal that phone resistance causes mixed psychological and physiological consequences.

As expected, people who resisted their phone were less entertained than those who used their device. This finding suggests that the mobile phone can facilitate perceived psychological benefits relative to self-control with the device. Participants also perceived more concentration difficulty and more mind wandering when they were without their device relative to when they used their phone. Reported levels of concentration difficulty were attenuated, however, when participants had the device in front of them but resisted it. Together, these results suggest that the relationship between self-control and mobile phones is complex and nuanced. On the one hand, a positive outcome of mobile phone resistance is decreased perceived concentration difficulty relative to sitting alone without external stimulation. On the other hand, people are less entertained when resisting the phone versus using the device.

Why can the presence of the mobile phone lead to a perceived improvement in concentration, even if the device is resisted? Prior theoretical work on self-control suggests that the saliency of a temptation triggers the motivation, attention, and value systems of an individual [15]. People see an object, understand what it provides, and its presence allows the individual to reflect on what it means to them [9]. Most people believe that phones are valuable (e.g., for social connection, to pass the time), and having the phone present offers something to think about compared to an unadorned room that does not provide this cognitive stimulus. Campbell and colleagues [26] also argue that having the phone offers cognitive reminders about “the potential” for mediated communication if needed (p. 178). Therefore, access to the mobile phone was more comforting and reduced perceived attention challenges than not having the device. The phone, when resisted, may have been a cognitive aid, providing a psychological experience similar to other cases where participants had a topic to think about and reported better focusing outcomes [10]. Future research should continue to probe the motivational and attentional processes of self-control, while also evaluating how the mobile phone can change psychological responses as well [55].

In light of these unexpected findings, we examined other literature to provide perspective for the idea that the presence of the phone can concentrate the mind. In an experiment, Rieger and colleagues [56] had participants color-code combinations of letters in a word, a task that becomes very tiring for participants over time. Participants who used their mobile phone in a subsequent waiting period reported increased levels of autonomy and control over their experience than participants who did not use their phone [56]. The authors argue that the mobile phone can be a “security blanket” and aid in recovery after mental fatigue. Using the phone provides comfort that the information on the device is secure (e.g., social connections, personal information, other valuable data) and it reinforces a sense of control that people have their social and professional worlds in their own hands [26,27]. Therefore, we believe that at least having the mobile phone available improved perceived concentration abilities because the device remained accessible and brought participants psychological comfort. In contrast with other work that suggests the mobile phone may have interpersonal consequences for conversation dynamics [7], our data suggests that there may be perceived intrapersonal benefits for those who at least have the phone in front of them relative to those who do not. The experience also affected how the participants’ body responded to temptation, as arousal levels were significantly lower for those who resisted the device relative to those who were without the phone toward the end of the six-minute experimental period. Future work should identify if the phone is special in focusing the mind or if other media experiences have similar characteristics.

### Replication and extension

With an increased focus on replication in the social sciences, this preregistered study reports positive replications for the Wilson and colleagues [11] entertainment, concentration difficulty, and mind wandering effects that occurred between participants in the no phone and phone use conditions. Their design was also extended by assessing physiological responses to temptation. Self-report measures and physiological responses do not always provide consistent signals (see [39]), and our investigation provides a nuanced understanding of how aloneness, self-control, and phone use may affect how people think and feel.

The skin conductance data revealed that arousal levels were consistent across conditions through the first three minutes of the study. After three minutes, participants who resisted their mobile phone experienced a steep decline in excitation while participants who were without their phone experienced an increase in arousal. Although speculative, these trends suggest that it may take time for participants to settle into their experience and physiologically respond to being with or without media. Eventually, people who were without their phone displayed heightened arousal responses, with increased skin conductance levels compared to those who at least had the mobile phone in their presence.

The prior perspective is consistent with research that has observed psychological responses diverge over time when people are with or without their phone. Cheever and colleagues [57] randomly assigned low, medium, and high frequency cell phone users to either turn off their device and keep it out of sight, or the device was removed and exchanged for a claim ticket while filling out surveys. The authors measured anxiety at three points, each twenty-minutes apart. High frequency users who did not have their phone reported increased anxiety across all three time periods, moderate users reported increased anxiety between the first two time-blocks but not the second and third time-blocks, and low frequency users showed no change in reported anxiety over time and generally reported the lowest anxiety overall. Though it is unclear how increased levels of arousal map on to direct psychological constructs in our study, future research should explore the connection between arousal responses and their psychological correlates.

It is also important to consider why certain effects failed to replicate or provide a reliable signal in our experiment. First, we observed that participants across conditions did not experience overall enjoyment differences. It is possible that participants in the phone use condition believed that their experience was similar to a waiting room period. Waiting rooms are not particularly arousing and may encourage the adherence to social norms [58]. With mobile media, norms are often updated and revised since they are context-dependent [59]. Therefore, if participants treated the phone use condition as an everyday experience like a waiting period (e.g., students checking their phone before class), perhaps this failed to amplify their enjoyment because it was a normative activity. This possibility should be explored in future qualitative and quantitative research on mobile phone use and social norms.

Second, consistent with prior work [10,11,18–21], we proposed that periods of free thinking can be less positive for people (e.g., they associate with more concentration difficulty and mind wandering) compared to phone use, but failed to observe negative affect differences on the PANAS scale. Why did this null effect emerge? One possibility is that the period of free thinking was not generally negative and instead, it only affected certain aspects of how people thought and felt (e.g., it increased their perceived concentration and focusing challenges). This idea is consistent with Alahmadi and colleagues [10], who suggest that participants generally do “not *hate* being alone with their thoughts,” but they may face psychological and physiological challenges related to aloneness more so than participants who have something to do (p. 546). We believe that these findings show the sensitivity and potential boundary conditions of the “just thinking” phenomenon. It is not unequivocally negative to be alone with one’s thoughts, but in certain cases compared to using or exercising self-control with the mobile phone, aloneness may cause perceived focusing challenges.

Finally, exercising self-control with the mobile phone failed to worsen psychological and physiological responses compared to being without the phone or using the device. In fact, exercising self-control and at least having access to the device improved perceived focusing abilities and reduced arousal levels over time. This result is reasonable if participants in the phone resist condition internalized their instructions as a rule-following task (e.g., “*We would like you to entertain yourself with your thoughts*, *but please do not use your phone for the duration of the experiment*,” see OSF) and were therefore extrinsically motivated to not use their phone. This idea may be connected to the observation that only one subject touched the phone (but did not use it) in the phone resist condition. Intrinsic and extrinsic motivation play a crucial role in experiences with media [60] and self-control [61], and future research should discover how increased intrinsic motivation to resist the phone may lead to downstream psychological and physiological effects.

After considering the significant and non-significant effects together, there are several take-aways from this study. First, the results are generally consistent with other evidence suggesting that periods of free thinking are not perceived as entirely negative [10,30,31], as participants often self-reported psychological effects that hovered around the midpoint. When measurements were more toward the extremes, such as the case of concentration difficulty, participants typically reported concentration ease rather than a challenge (typical scores below the midpoint: *Mdn* = 3). Only after comparing free thinking to a period of external stimulation or self-control did a less positive experience emerge for those without their device. Therefore, it is important to consider free thinking not as a challenge in absolute terms, but as a cognitive challenge relative to other experiences that may occur with media (e.g., use, resistance).

A second take-away suggests that free thinking is generally less positive for attention variables (e.g., concentration difficulty and mind wandering), but not enjoyment. One possible explanation for the null enjoyment finding is that people may have felt it was socially undesirable to report enjoyment with media (for related evidence, see [62]). Participants might have underreported their feelings with the mobile phone because of pressures to appear like they were not dependent on technology for enjoyment. Social desirability biases are important to consider in many self-report evaluations (e.g., [63]) and should be accounted for in future research.

Third, the concentration data are theoretically interesting because they suggest that thinking aids can also be represented by physical objects. In prior work [10,18], giving participants a thinking aid (e.g., topics that can focus the mind, such as one’s family) led to perceived psychological benefits versus not having a thinking aid. Our evidence offers that thinking aids can also be media (e.g., the mobile phone), though it is unclear how physical versus thought-derived thinking aids differ in their ability to reduce perceived concentration challenges. This is an opportunity for future research to discover boundary conditions of the prior effects.

Together, when people are without the mobile phone, their mind and body tend to feel attention challenges but generally not enjoyment. These results offer an important perspective on the psychological and physiological limits of free thinking relative to self-control and mobile phone use. Continuing to probe the conditions that facilitate or curb such effects, and testing a diversity of media, is a worthwhile effort for future work.

### Limitations and future research

There are several limitations of this study worth resolving. First, it is unclear what mobile phone activities were performed in the phone use condition because the experiment failed to collect phone activity data. Therefore, future work should identify if mobile content or activity moderates the reported effects. For those who resisted the phone, it is also unclear what they thought they were resisting. Future studies could ask people what activities they felt tempted to perform with their phone (e.g., talk to friends, surf the web).

Participants in the phone use condition were instructed to not text or call others during the experimental period and this instruction may represent a form of resistance. Prior work suggests that notifications and other social reminders can undermine focus on a particular task [64], leading us to institute this restriction and to remain compliant with our replication effort of prior procedures [11]. Further, people in the phone use condition may have had a clearer task (e.g., entertain themselves with their phone) than people in the no phone or phone resist conditions. Task demand differences, therefore, may have affected how people thought or felt during the experience and they should be mitigated in future research. It is also important to note that participant tasks in the phone use condition were self-generated (e.g., participants chose what was personally entertaining), which may differ across individuals. We attempted to constrain the activities that people could perform on their phone by instructing participants to not call or text [11], though future research should provide explicit activity instructions to ensure task consistency (e.g., only work-related tasks such as answering email).

Future versions of this experiment should also extend the length of being without, resisting, or using the mobile phone (for additional commentary, see [65,66]). Our evidence suggests that participant EDA begins to diverge after approximately three minutes and it is unclear if EDA converges at a future time marker as well. We observed that participants in the phone resist and no phone conditions have opposite excitation responses between minutes three and six. Future experiments should examine the mechanisms at play during this period to understand the processes that contribute to such arousal differences.

Future research should expand the type and number of measures that are used in similar studies. Including measures that probe the degree to which people felt a desire to use their phone, or how much their self-control goals were in conflict, would be important to have baseline gauges of willpower and desire. We expect that those who experience a large desire to use their phone (or feel conflicted about their self-control goals) may face amplified psychological and physiological consequences relative to those who have a small desire to use their phone (or feel unconflicted about their self-control goals). Our data suggest, however, that those who are without their phone still report different cognitive outcomes and physiological changes than those who are told not to use their device but have it in front of them.

Finally, having a multidimensional and multi-item view of concentration difficulty or mind wandering (e.g., [22]) can help to understand the intricacies of this phenomenon, which are inherently complex [23,24]. Interviewing participants to understand how they also conceptualize mind wandering, either as a generally negative phenomenon or something that can be meditative under certain conditions, may provide a nuanced perspective of this psychological construct. Relatedly, participants did not receive an instruction for how mind wandering should be interpreted. Our conceptualization, which suggests that concentration difficulty and mind wandering are related constructs and reflect poor attentional control for tasks that involve free thinking [10,23], should be better communicated to participants to ensure consistent interpretations. Prior work suggests that participant understanding of mind wandering might not be uniform [67,68]. Therefore, other versions of this study should clarify mind wandering and measure it in multiple ways.

## Conclusion

The data from this study suggest that people report less positive psychological consequences (e.g., more concentration difficulty, more mind wandering) when they are without their phone relative to when they use or resist the phone. Skin conductance data were largely consistent with this pattern and suggest that resisting the phone is less arousing than being without the device. These data partially replicate and also extend empirical evidence suggesting that “just thinking” is less positive than having something to do. Self-control with the mobile phone is an important research area and future work should continue to investigate the social, psychological, physiological, and design factors that influence one’s ability to resist temptation with technology.

## Supporting information

S1 Table Multiple comparisons tests across measures. In S1 Table, all *p*-values are Bonferroni corrected. The boring item was reverse-scored for the enjoyment index, only. Superscripts denote a higher mean for that condition relative to the adjacent condition, for the same dependent variable.
(XLSX)
